# Antimicrobial and Antibiofilm Activity of a Recombinant Fragment of β-Thymosin of Sea Urchin *Paracentrotus lividus*

**DOI:** 10.3390/md16100366

**Published:** 2018-10-02

**Authors:** Angelo Spinello, Maria Grazia Cusimano, Domenico Schillaci, Luigi Inguglia, Giampaolo Barone, Vincenzo Arizza

**Affiliations:** 1 CNR-IOM-DEMOCRITOS c/o International School for Advanced Studies (SISSA), Via Bonomea 265, 34136 Trieste, Italy; aspinello@sissa.it; 2 Dipartimento di Scienze Biologiche, Chimiche e Farmaceutiche, Università di Palermo, Via Archirafi 18, 90123 Palermo, Italy; domenico.schillaci@unipa.it (D.S.); luigi.inguglia@unipa.it (L.I.); giampaolo.barone@unipa.it (G.B.); vincenzo.arizza@unipa.it (V.A.)

**Keywords:** AMP (antimicrobial peptides), biofilm, *Staphylococcus aureus*, *Pseudomonas aeruginosa*, thymosin, *Paracentrotus lividus*, molecular dynamics

## Abstract

With the aim to obtain new antimicrobials against important pathogens such as *Staphylococcus aureus* and *Pseudomonas aeruginosa*, we focused on antimicrobial peptides (AMPs) from Echinoderms. An example of such peptides is Paracentrin 1 (SP1), a chemically synthesised peptide fragment of a sea urchin thymosin. In the present paper, we report on the biological activity of a Paracentrin 1 derivative obtained by recombination. The recombinant paracentrin RP1, in comparison to the synthetic SP1, is 22 amino acids longer and it was considerably more active against the planktonic forms of *S. aureus* ATCC 25923 and *P. aeruginosa* ATCC 15442 at concentrations of 50 µg/mL. Moreover, it was able to inhibit biofilm formation of staphylococcal and *P. aeruginosa* strains at concentrations equal to 5.0 and 10.7 µg/mL, respectively. Molecular dynamics (MD) simulations allowed to rationalise the results of the experimental investigations, providing atomistic insights on the binding of RP1 toward models of mammalian and bacterial cell membranes. Overall, the results obtained point out that RP1 shows a remarkable preference for bacterial membranes, in excellent agreement with the antibacterial activity, highlighting the promising potential of using the tested peptide as a template for the development of novel antimicrobial agents.

## 1. Introduction

The onset of multidrug-resistant Gram-positive and Gram-negative bacterial strains has made most of the current antibiotics less effective, making the research of novel therapeutic strategies against pathogens an urgent topical issue [1]. Furthermore, even if conventional antibiotics can be effective against free living bacterial cells, there are few agents that effectively target the pathogens in a biofilm form. The biofilm is a three-dimensional microbial community that grows on both biological and abiotic surfaces. Opportunistic pathogens, such as staphylococcal strains and *Pseudomonas aeruginosa,* show a great ability to produce biofilms that prevent infected wounds from healing, rendering the treatment extremely challenging [2]. Unfortunately, the introduction in therapy of new molecules effective against *P. aeruginosa* and staphylococcal strains is extremely slow. Additionally, the treatment of biofilm-associated infections is complicated because microbial biofilms show a multifactorial antibiotic resistance linked to their growth as a community [3]. The discovery of anti-infective agents active against both forms of growth, planktonic and biofilm, represents a fundamental goal for an effective control of infections [4,5]. This has stimulated the research for antibiotic alternative strategies in the struggle against pathogens. Many antimicrobial peptides (AMPs) show a high specificity for prokaryotes and a low toxicity for eukaryotic cells and, due to their mode of action, the development of resistance is considered less probable. It is well known that the marine invertebrates are a good source of AMPs [6], and a variety of peptides with antimicrobial properties have been isolated from the Echinodermata phylum [7,8]. We recently found and described three peptides, fragments of a β-thymosin of *Paracentrotus lividus*, the sea urchin from Mediterranean Sea [9]. In particular, we focused on the smallest 11 amino acid peptide (9–19), whose molecular weight was 1251.7, that we called Paracentrin 1 (SP1, EVASFDKSKLK). We demonstrated a broad range of activity against planktonic and biofilm forms of representative pathogens, like staphylococcal strains and *P. aeruginosa,* but we found Minimum Inhibitory Concentration (IC)values in the order of mg/mL [10]. Subsequently, we compared the amino acid sequence of SP1 with the corresponding fragment 9–19 (EIEKFDKSKLK) of human thymosin β4 (Tβ4). We observed, using molecular dynamics (MD) simulations, that the conformations adopted by these two peptides in a physiological environment are very similar and, then, their interaction with model membranes and biological activities *in vitro*, with MIC values ranging from 12.5 to 6.2 mg/mL [11]. 

In order to improve the biological activity of SP1 we obtained, by recombination, the recombinant Paracentrin 1 (RP1), being 22 amino acids longer in the amino terminal region in comparison with SP1 (MSGSHHHHHHGSSGENLYFQSLEVASFDKSKLK, with SP1 portion highlighted in bold). In the present study, we compared the structure and dynamics of RP1 with the chemically synthesised SP1. The interaction of RP1 with two membrane models in silico, and the antibacterial and anti-biofilm activity in vitro, were also evaluated. Two lipid bilayers were used in this study: POPC (1-palmitoyl-2-oleoylphosphatidylcholine) and POPC: POPG (1-palmitoyl-2-oleoylphosphatidylglycerol) (2:1). These models were chosen in order to mimic the mammalian and bacterial cell membrane, respectively. Additionally, the antibacterial and antibiofilm activities against a staphylococcal reference strain *S. aureus* ATCC 25923 and *P. aeruginosa* ATCC 15442 are reported.

## 2. Results

### 2.1. Antibacterial Activity of RP1

RP1 was tested in vitro at concentrations ranging from 100 to 0.75 µg/mL against two reference bacterial strains, *S. aureus* ATCC 25923, and *P. aeruginosa* ATCC 15442. The antibacterial activity, expressed as MICs (minimum inhibitory concentrations), is reported in Table 1. RP1 is able to interfere with microbial growth of all tested strains at MIC concentrations of 50 µg/mL. It is interesting to highlight that RP1 resulted in being 250 times more active against *S. aureus* ATCC 25923 and *P. aeruginosa* ATCC1 15442 than SP1 (MIC of 12.5 mg/mL against abovementioned bacterial strains) [10], and comparable with the MIC of LL-37 a human AMP derived from cathelicidin hCAP-18.

### 2.2. Interference with Biofilm Formation 

The ability of RP1 to inhibit biofilm formation of staphylococcal reference strains, as *S. aureus* ATCC 25923 and *P. aeruginosa* ATCC 15442, was tested at concentrations lower than the MIC observed against planktonic form. The values of biofilm inhibition concentration, BIC_50_, in interfering with biofilm formation, were found to be of 5.0 µg/mL against *S. aureus* ATCC 25923 and 10.7 µg/mL against *P. aeruginosa* ATCC 15442; see Table 2. Remarkably also, in this case, we observed a considerable improvement of the activity, being 1000 times more active as biofilm inhibitor than SP1 (whose BIC_50_ were 0.3 and 1.5 mg/mL against the abovementioned pathogens). LL-37, tested for comparative purposes, showed a BIC_50_ of 1.6 µg/mL or 12.9 µg/mL, respectively, against the staphylococcal strains or *P. aeruginosa* ATCC 15442. 

### 2.3. Molecular Dynamics of RP1

**Figure 1:**
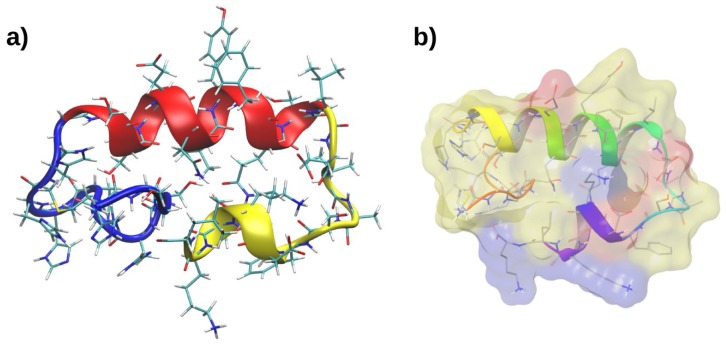
RP1 folded structure. The structure of RP1 is divided in three parts, which are shown in three different colours: blue, red, and yellow, respectively, for RP1-1, RP1-2, and RP1-3 (**a**). Positively (blue) and (**b**) negatively (red) charged residues are shown.

The novel recombinant peptide investigated in this work, RP1, has the following amino acid sequence: MSGSHHHHHHGSSGENLYFQSLEVASFDKSKK. Due to the longer sequence of RP1 in comparison with the previously investigated SP1 [10], we have generated a starting peptide conformation using the PEP-FOLD webserver [12]. A 500 ns MD simulation was performed on the lowest energy model, in order to relax the structure in a physiological environment. This conformation proved to be stable during the whole simulation and the most representative structure was extracted using a cluster analysis. According to the secondary structural motifs, we can ideally divide RP1 in three sections (see Figure 1). The first portion (RP1-1), ranging from Met1 to Gly11 (Figure 1a blue), is composed mainly by histidine residues, and it remains mostly unstructured at the end of the MD simulation. The second part represents the main peptide core (RP1-2), and it is formed by an α-helix made by residues Ser12–Leu22 (Figure 1a, red). The third portion of RP1 (RP1-3), from Glu23 to Lys33, corresponds to the SP1 peptide having a partially unfolded structure, stabilised by intramolecular hydrogen bonds with the main helical core (Figure 1a, yellow). The most representative RP1 conformation is constituted by an asymmetric distribution of charges between the less polar α-helical core RP1-2 and the two more polar RP1-1 and RP1-3 sequences (see Figure 1b). RP1-3 possesses the highest number of charged residues (one Glu, one Asp, and three Lys) while the remaining part is still slightly polar, and has only one charged residue (Glu15).

### 2.4. Interactions with Membrane Models In Silico

**Figure 2:**
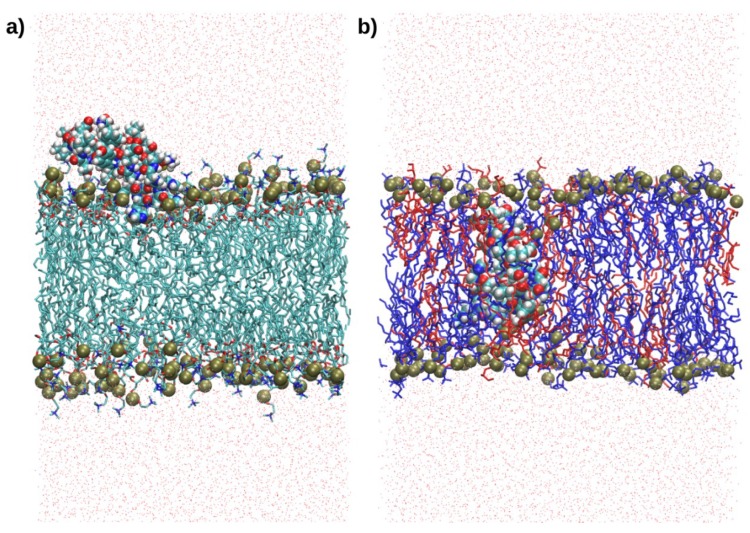
The selective interaction between RP1 and the mammalian and bacterial membrane models. Two representative snapshots showing the interaction of RP1 with (**a**) the POPC and (**b**) the POPC (blue)/POPG (red) membrane models. RP1 and phosphorous atoms are shown in van der Waals representation.

**Figure 3:**
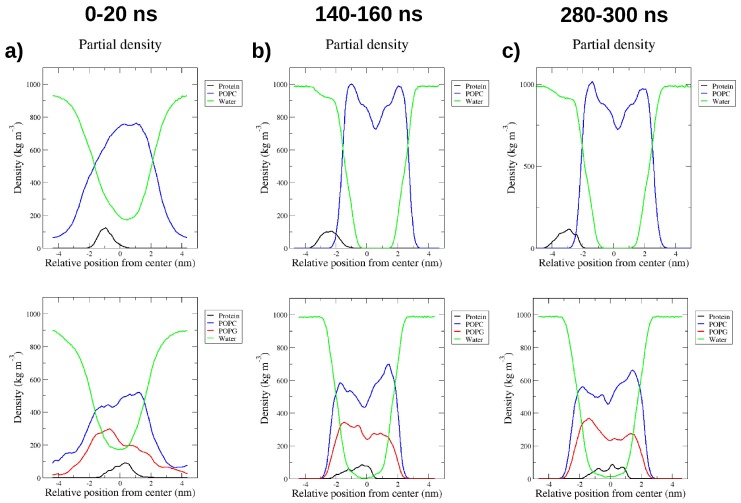
RP1 mass density profiles. Mass density profiles calculated in three representative windows, (**a**) 0–20 ns, (**b**) 140–160 ns, and (**c**) 280–300 ns, for the MD simulations of RP1 in POPC (top) and in POPC/POPG (bottom) membrane models.

**Figure 4:**
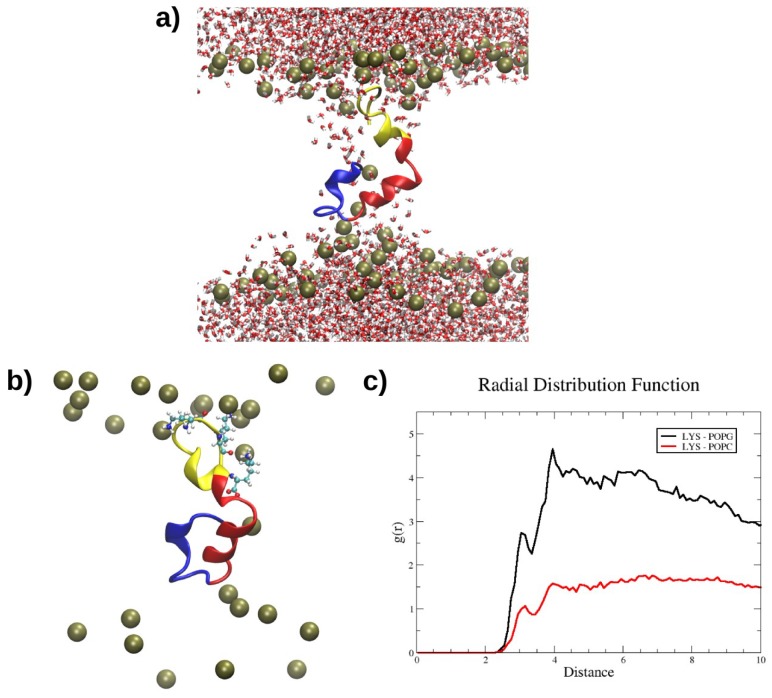
Preferential interaction of the positively charged Lys residues of RP1-3 with the negatively charged POPG heads. Snapshot showing the membrane defect formed in the POPC/POPG simulation. (**a**) The interaction between Lys residues and the phosphate groups of the POPG lipids (P atoms are shown in van der Waals representation). (**b**) Radial distribution function showing the distance (Å) between the Lys residues and the phosphorous atoms of POPG (black line) and POPC (red line) lipids (**c**).

The interaction of RP1 with two bilayer models, POPC and POPC/POPG (2:1), which mimic the mammalian and bacterial membranes, respectively, was investigated by MD simulations. The most representative RP1 conformation was used as a starting structure for the study of the interaction with the membranes. In particular, 300 ns MD simulations were performed for both membrane models using the “minimum bias” method, [13] in which the lipids self-organise spontaneously in ordered bilayers in reasonable time-scales, i.e., less than 100 ns. Remarkably, while RP1 binds only superficially to the mammalian POPC model, it suddenly inserts and remains deeply buried inside the bacterial POPC/POPG model, as shown in Figure 2, until the end of the MD simulation. The position of RP1 and the evolution of the forming bilayer are shown using density profiles taken at different simulation times (Figure 3). At the beginning of the POPC-RP1 MD simulation, water and lipids are almost homogeneously mixed and, after 100 ns, the lipid bilayer is formed. The charged SP1 portion of the peptide, RP1-3, preferentially interacts with the POPC polar heads until the end of the simulation, while the remaining RP1 residues are still solvated (Figure 3c top). Remarkably, the POPC/POPG-RP1 simulation shows a different behaviour. After 140 ns, two almost symmetrical leaflets are formed showing similar lipid composition (Figure 3b,c bottom). RP1 is localised almost in the centre of the membrane, deeply affecting the shape of the bilayer and remaining stably bound until the end of the simulation. The same RP1 interaction with the POPC/POPG membrane model was confirmed in another 300 ns replica. Moreover, while the bilayer is formed, water is not completely excluded from the hydrophobic centre, suggesting the presence of a membrane defect induced by RP1. In order to check the stability of the defect, two annealing cycles (as described in the Materials and Methods section), each followed by 100 ns of MD simulation, were performed, for a total simulation time of about 500 ns. The defect formed by the presence of RP1 was not healed after both annealing steps (see Figure 4a), suggesting that this is not an artefact introduced by the simulation, but real membrane damage induced by the presence of RP1 inside the bacterial model. This remarkable selectivity seems to be driven by the electrostatic interactions among the positively charged Lys residues of RP1-3 with the negatively charged POPG heads (Figure 4b), as clearly shown by the radial distribution function, g(r), between Lys residues and the phosphorous atoms of the POPC and POPG lipids (Figure 4c). Hence, RP1-3 acts as an anchor, while the first two, more hydrophobic RP1 portions, RP1-1 and RP1-2, remain deeply buried inside the newly formed membrane.

## 3. Discussion

In this study, we prepared a recombinant new peptide, RP1, deriving from SP1, an antibacterial peptide present in *P. lividus* coelomocytes [10]. The structure of RP1 is significantly different from the chemically synthesised SP1. In fact, the addition of the RP1-1 and RP1-2 moieties to the SP1 original structure (represented here by the RP1-3 portion, see Figure 1) enriches the novel recombinant peptide with important structural features shared by common AMPs: (i) an α-helix structural core; (ii) a net positive charge that favours electrostatic attractions towards the negatively charged microbial membranes; and (iii) an amphipathic organisation, that confers to RP1 a stronger bilayer interaction, especially with bacterial membrane models. As a consequence, RP1 has shown a remarkable improvement of the antimicrobial and antibiofilm activity in comparison to SP1 (which resulted to be 250 and 1000 times higher, respectively) and an activity in vitro against tested pathogens similar to human host defence peptide LL-37. The amphipathic α-helical antimicrobial peptides are produced by organisms that are evolutionarily quite distant, ranging from protostomes such as insects [14,15,16] to deuterostomes such as echinoderm [17], tunicates [18], and vertebrates [19,20,21]. They have evolved to function in many different environments, such as the haemolymph of insects [22], amphibian skin secretions [23,24,25], gastric mucosa and intestinal epithelia, mammalian phagocytic vacuoles, wound and blister fluids, and epithelia [26,27,28]. In this work, we have performed MD simulations with the aim to provide the molecular mechanism by which the title peptide exerts its biological activity. Remarkably, our simulations have highlighted a preferential interaction of RP1 with the bacterial membrane model, due to the electrostatic attraction among the negatively charged POPG lipids and the positively charged Lys residues of RP1-3, being in excellent agreement with the antibacterial and the antibiofilm activity experimentally observed. Furthermore, such peptides could, in principle, also hit metabolically low active bacterial cells, intrinsically resistant to conventional antibiotics. 

## 4. Materials and Methods 

### 4.1. Paracentrin 1 Gene Cloning

The nucleotide sequence of the sea urchin Paracentrin 1 peptide was cloned in a pFastBac™-Dual vector (Life Technologies Italia, Monza, Italy)) using the Bac-to-Bac^®^ N-His TOPO^®^ cloning kit (Life technologies). In particular, two partially complementary oligonucleotides were synthesised by BMR Genomics Custom Primer Service, Padova, Italy) (For: 5’-GAAGTTGCATCTTTCGACAAGTCGAAACT-3’, Rev: 5’-TTACTTAAGTTTCGACTTGTCGAAAGATG-3’) and used as primer and template in a PCR reaction with the Platinum Pfx DNA Polymerase (Life Technologies) (25 cycles: 94 °C for 15 s, 52 °C for 30 s, 68 °C for 45 s, and a final extension at 68° C for 10 min.) The oligonucleotides were designed in order to include the Paracentrin 1 gene sequence and a stop codon at the end of the gene.

### 4.2. Peptide Expression

Recombinant bacmid was generated in DH10Bac™ cells using the Bac-to-Bac^®^ N-His TOPO^®^ (Life technologies) and extracted using Ni-NTA Purification system (Invitrogen). In particular, *Spodoptera frugiperda* (Sf9) cells (Life Technologies) were cultured in suspension flasks on a rotating platform (110 RPM at 26 °C) using an incubator shaker and Sf-900 II media following the supplier’s manual. Recombinant progeny 1 baculovirus (RPB1) was produced in monolayer cultures by transfecting 5 mL Sf9 cells (1.0 × 10^6^ cells/mL grown in 25 cm^2^ flasks), and was harvested after 6 days, aliquoted, and stored at −80 °C. Recombinant progeny 2 baculovirus (RPB2) virus was generated by infecting Sf9 suspension cultures (1.5 × 10^6^ cells/mL) with RPB1 virus (400 μL/100 mL cells). The supernatant from this culture (RPB2) was harvested 72 h post-infection, and 3 mL was used to infect 600 mL of Sf9 suspension culture (1.5 × 10^6^ cells/mL), thus generating a recombinant progeny 3 baculovirus (RPB3) virus 72 h post-infection. The baculovirus present in this culture (RPB3) was quantified using the kit BacPak qPCR titration kit (Clontech Takara Bio USA, Inc.) and 2 L of Sf9 cells (1.0 × 10^6^ cells/mL) were infected at a MOI (multiplicity of infection) of 5 for protein production. 

For His-tagged protein production, cells were grown in suspension for 48 h, and the cell pellets was used for protein extraction according to the Ni-NTA Purification system (Invitrogen) protocol. An aliquot of the eluate was loaded on a Sepharose 100 column, pre-equilibrated in 10 mM sodium phosphate buffer.

MSGSHHHHHHGSSGENLYFQSLEVASFDKSKLK

### 4.3. Microbial Strains 

*Staphylococcus aureus* ATCC 25923 and *Pseudomonas aeruginosa* ATCC 15442, the reference strains in official tests for antibacterial evaluation in vitro (UNI EN European Standard), were used in this study.

### 4.4. Minimum Inhibitory Concentrations (MICs)

MICs were determined by a previously described micromethod [10]. Briefly, a series of solutions were prepared with a range of concentrations from 100 to 0.75 µg/mL (obtained by two-fold serial dilution). The serial dilutions were made in tryptic soy broth (TSB) (VWR International, Leuven) in a 96-wells plate, starting from a stock solution of 1 mg/mL in NaCl 0.9% *w*/*v*. To each well, 10 μL of a bacterial suspension from a 24 h culture containing ~10^6^ cfu/mL was added. The plate was incubated at 37 °C for 24 h; after this time, the MICs were determined by a microplate reader (Glomax Multidetection System TM297 Promega, Milano Italy) as the lowest concentration of compound whose OD, read at 570 nm, was comparable with the negative control wells (broth only, without inoculum). We also tested the known active peptide, LL-37, for comparative and quality control purposes. Each assay was performed in triplicate and repeated at least twice.

### 4.5. Evaluation of Biofilm Formation and Biofilm Prevention Assay

The bacterial reference strains were tested for their ability to form biofilms. The evaluation assay was previously described elsewhere [29]. Briefly, *S. aureus* ATCC 25923 and *P. aeruginosa* ATCC 15442 were grown, diluted, and wells were washed as described by [30]. The plates were air-dried at 37 °C, and each well was filled with 100 μL of TSB supplemented with several concentrations, ranging from 12.5 to 0.2 μg/mL, of RP1 or LL-37 at sub-MIC concentrations, except in the case of positive controls. The plates were incubated at 37 °C for 24 h; after this incubation time, the medium was removed, the plates were air-dried and, then, a crystal violet solution (0.1%) was added to each well, followed by incubation for 15–20 min. The plate was then washed three times with water, and the crystal violet was dissolved in 150–200 μL of ethanol by pipetting up and down. The plate was read at 570 nm using a microplate reader (Glomax Multidetection System Promega). Biofilm inhibitory concentration (BIC_50_), that is, the concentration at which the percentage of inhibition of biofilm formation (see below) is equal to 50% were obtained by comparing the optical densities (ODs) of control wells with that of the sample wells, and the value was calculated by using a linear regression graph in Excel. Each assay was performed in triplicate and repeated at least twice. 

The percentage of inhibition was calculated through the formula

% of inhibition = ((OD growth control − OD sample)/OD growth control) × 100(1)

### 4.6. Molecular Dynamics Simulations

The PEP-FOLD webserver [12] was used to obtain a folded structure of RP1 and the lowest energy model was taken as a starting point for subsequent simulations. The stability of this conformation in physiological conditions was investigated, in silico, by molecular dynamics (MD) simulations, following reported procedures [31]. The interaction of RP1 with two membrane models was studied using the “minimum-bias” method, [13] in which disordered lipids self-organise spontaneously in the presence of AMPs. The software packmol [32] was used to generate the starting configuration. Peptides were placed in the centre of a cubic box having sides of 12 Å. Into the box were added 128 POPC for the mammalian model, 86 POPC and 42 POPG for the bacterial model, as well as 7500 water molecules. The Amber99SB-ILDN force field [33], implemented in the GROMACS 4.6.5 software package [34], was used in combination with the Slipids (Stockholm lipids) force field for lipids [35,36]. Pressure coupling was applied anisotropically, using a Parrinello–Rahman barostat with a reference value of 1 bar [37]. First, a short 100 ps equilibration was performed in order to achieve the target density of the system, followed by 300 ns production runs. MD simulations were replicated. In the case of the POPC/POPG, model MD simulations were followed by annealing cycles in which the temperature was increased from 300 K to 375 K in 2 ns and, again, decreased to 300 K in 50 ps, as previously reported in the literature, in order to heal membrane defects [38]. Afterwards, another 100 ns simulation was performed at 300 K. Figures and plots were obtained by the VMD software [39]. Density profiles were calculated with the GROMACS tool g_density. Clustering analysis was performed by g_cluster tool, also included in the GROMACS package.

## 5. Conclusions

Considering the recent dramatic increment of multidrug-resistant microbial pathogens and the urgent need for novel therapeutic strategies, our results lay the foundation for the development of novel AMPs using RP1 scaffold as a potential and effective template.

## Figures and Tables

**Table 1:** Antibacterial activity, in vitro, of RP1 and LL37.

Reference Strains	MIC (µg/mL)	
	RP1	LL-37
*S. aureus* ATCC 25923	50	50
*P. aeruginosa* ATCC 15442	50	50

**Table 2:** Inhibition of biofilm formation.

Reference Strains	BIC (µg/mL)	
	RP1	LL-37
*S. aureus* ATCC 25923	5.0 ± 0.3	1.6 ± 0.04
*P. aeruginosa* ATCC 15442	10.7 ± 0.7	11.9 ± 0.9
